# Clinicians’ Perceptions About Institutional Factors in Moral Distress Related to Potentially Nonbeneficial Treatments

**DOI:** 10.1001/jamanetworkopen.2025.16089

**Published:** 2025-06-16

**Authors:** Teva D. Brender, Julia K. Axelrod, Sofia Weiss Goitiandia, Jason N. Batten, Elizabeth W. Dzeng

**Affiliations:** 1 Department of Medicine, University of California, San Francisco; 2 Division of Hospital Medicine, Department of Medicine, University of California, San Francisco; 3 Division of Cardiothoracic Anesthesiology, Department of Anesthesiology and Perioperative Medicine, University of California, Los Angeles; 4 Philip R. Lee Institute for Health Policy Studies, University of California, San Francisco; 5 Cicely Saunders Institute of Palliative Care, Policy, and Rehabilitation, King’s College London, London, United Kingdom

## Abstract

**Importance:**

Clinicians commonly experience moral distress related to potentially nonbeneficial life-sustaining treatments (LST). Hospitals’ institutional culture (eg, shared beliefs, values, and practices), structures (eg, policies, practices, resource allocation), and societal-level factors (eg, national culture, local and national policies, medical hierarchies) may contribute to moral distress related to potentially nonbeneficial LST.

**Objective:**

To investigate clinicians’ perspectives on how hospitals’ institutional culture and structures might exacerbate, prevent, or mitigate the influence of societal factors contributing to moral distress related to potentially nonbeneficial LST.

**Design, Setting, and Participants:**

This qualitative study used comparative ethnographic methods. Semistructured, in-depth interviews were conducted between February 2018 and June 2022 at 4 West Coast academic hospitals selected for their varying intensities of end-of-life care. Interview participants were hospital-based clinicians (eg, nurses, physicians), hospital leaders (eg, unit nursing and medical directors), and administrators with differing clinical backgrounds and professional responsibilities. Data were analyzed in 2 phases, from January 2019 to December 2022 and from June to September 2024.

**Main Outcomes and Measures:**

Clinicians were asked about their hospitals’ institutional culture and structures and their relationship to clinicians’ experiences of moral distress related to potentially nonbeneficial LST in end-of-life care.

**Results:**

A total of 122 interviews were conducted (75 physicians [61%]; 22 nurses [18%]; 6 advanced practice clinicians [6%]; 68 [56%] women; mean [range] age, 42 [27-74] years). Respondents felt hospitals’ institutional culture and structures could exacerbate moral distress. Respondents reported that a hospital culture of health care consumerism influenced clinicians’, patients’, and families’ expectations for treatment intensity, contributing to morally distressing situations. Nurses and primary team physicians felt constrained by medical hierarchies, leading to perceptions of disempowerment and moral distress. Clinicians also reported that institutions lacked sufficient structures to support efforts to de-escalate potentially nonbeneficial treatments. However, respondents also reported that hospitals’ institutional culture and structures could prevent or mitigate moral distress. Respondents felt policies empowering clinicians across the medical hierarchy to participate in decision-making reduced moral distress. They reported that institutional resources could manage conflicts and provide emotional support when moral distress occurs. Furthermore, respondents felt that clinician-driven quality improvement initiatives and supportive hospital leaders could address hospitals’ institutional cultural and structural contributors to moral distress.

**Conclusions and Relevance:**

In this qualitative study, clinicians perceived that institutional factors affected their experiences of moral distress related to potentially nonbeneficial LST by exacerbating, preventing, or mitigating the influence of 3 societal factors: defaults of high-intensity treatments, health care consumerism, and medical hierarchies. These results have implications for developing tailored institutional-level interventions to address societal and institutional contributors to moral distress from potentially nonbeneficial LST.

## Key Points

**Question:**

What are clinicians’ perspectives on how hospitals’ institutional culture and structures can exacerbate, prevent, or mitigate experiences of moral distress related to potentially nonbeneficial life-sustaining treatments?

**Finding:**

In this qualitative study, 122 semistructured, in-depth interviews were conducted with hospital-based clinicians and administrators at 4 West Coast academic hospitals. Participants reported that hospitals’ institutional culture and structures affected clinicians’ experiences of moral distress by exacerbating, preventing, or mitigating the influence of societal factors, including consumerism, medical hierarchies, and defaults of high-intensity treatments.

**Meaning:**

These findings suggest health systems should consider tailored institutional-level interventions to address societal and institutional contributors to moral distress from potentially nonbeneficial life-sustaining treatments.

## Teaser

This qualitative study describes clinicians’ perspectives on how hospitals’ institutional culture and structures might exacerbate, prevent, or mitigate the influence of societal factors contributing to moral distress related to potentially nonbeneficial life-saving treatments.

## Introduction

Potentially nonbeneficial life-sustaining treatments (LST) (eg, mechanical ventilation, continuous kidney replacement therapy, vasopressors) may challenge clinicians’ ethical obligations of acting in patients’ best interests and doing no harm.^1,2^ Clinicians perceive that 20% to 40% of patients who are seriously ill receive potentially nonbeneficial LST near the end of life.^3,4,5^ Potentially nonbeneficial LST and other high-intensity treatments (eg, chemotherapy) are a significant source of moral distress for clinicians.^6,7,8,9,10,11,12,13^ Moral distress can be exacerbated if clinicians believe treatments are goal-discordant.^14^

Moral distress was first described by Jameton^9^ as occurring when clinicians believe they cannot act according to their ethical values due to societal, hierarchical, and institutional constraints. Alternative definitions of moral distress have since been proposed.^15^ We,^8,10^ and much of the clinical literature, use Jameton’s original formulation.^9^ When clinicians experience moral distress, they perceive negative effects on patient care.^11,12^ Clinicians who experience moral distress have worse physical and mental health,^13,16^ lower job satisfaction,^17^ and higher rates of burnout^18^ and are more likely to leave their profession.^19,20^ Moral distress may be an ethical root cause of burnout^21,22,23^ and a threat to health care systems, given health care worker shortages.^24^

Institutional and societal factors may exacerbate moral distress. Institutional factors, such as culture (eg, a hospital’s shared beliefs, values, and practices) and structures (eg, policies, practices, and resource allocation), include staffing shortages, high workloads, resource limitations, care rationing, and hospital ethical climate.^25,26,27,28,29,30^ Societal factors of interest to this study include national culture, local and national policies,^31,32^ and formal (eg, nurse-physician)^8,33,34^ and informal (eg, between-specialties) medical hierarchies.^35,36,37,38^ Less is known about how institutional factors may affect how societal factors contribute to moral distress related to potentially nonbeneficial LST.^30,39^

Strategies addressing moral distress related to potentially nonbeneficial LST have primarily focused on the individual clinician,^26^ such as mindfulness training,^12,40^ personal resilience,^22,41,42^ and debriefing.^8,43^ Yet an emphasis on individuals may overlook and divert attention from solutions targeting underlying institutional and societal contributors to moral distress.^26,44^

We previously described how hospitals’ institutional culture and structures shape default care trajectories toward high-intensity treatments near the end of life.^8,10,32,45^ This study used the same dataset to analyze root causes of moral distress.^30^ This study investigates how a hospital’s institutional culture and structures may exacerbate, prevent, or mitigate the influence of societal factors contributing to moral distress related to potentially nonbeneficial LST.

## Methods

This qualitative study was approved by the University of California, San Francisco, institutional review board. All participants provided written (before the COVID-19 pandemic) or verbal (during and after the COVID-19 pandemic) consent. We report our study using the Consolidated Criteria for Reporting Qualitative Research (COREQ) reporting guideline.

### Design

Interviews were conducted at 4 academic hospitals in California and Washington between February 2018 and June 2022. Hospitals were selected for their varying intensity of end-of-life care as characterized by the Dartmouth Atlas.^32,46^

### Data Collection

E.W.D., a hospitalist and sociologist, conducted semistructured, in-depth interviews. Interviews occurred in person until the COVID-19 pandemic, when interviews transitioned to video-conferencing. Interviews were audiotaped, transcribed, and anonymized. Interview participants were hospital-based emergency, internal medicine, and subspecialty clinicians (eg, nurses and hospital, critical, and palliative physicians), hospital leaders (eg, unit nursing and medical directors), and administrators with differing clinical backgrounds and professional responsibilities. Participants self-reported their demographic information, professional role, and years of experience. Race and ethnicity were collected as Asian, Black or African American, Hispanic, Middle Eastern, multiethnic, White, and other or no information provided (ie, individuals who chose not to disclose race or ethnicity). Race and ethnicity were collected as we aimed to maximize participant diversity and record demographic information. Interviewees were recruited through group and individual email solicitation and snowball sampling.^47,48^ The participation rate could not be calculated because persons were contacted through unsolicited emails, listservs, and direct solicitations.

All interviews used the same interview guide, with minor adaptations for different clinical roles.^32^ Interviews were open-ended and encouraged participants to discuss topics they considered most relevant. Themes and patterns developed from analysis of earlier interviews were probed in subsequent interviews. Recruitment occurred until theoretical saturation was reached, at which point interviews generated no additional insights.^49^

### Data Analysis

Data analysis and collection were performed concomitantly and iteratively. To develop an initial codebook, J.N.B., E.W.D., and others used thematic analysis on a subset of interviews, generating codes using line-by-line inductive and deductive analysis based on literature review and our group’s prior work.^8,10,28,32^ J.N.B. and others double-coded 20% of all the interviews, further refining the codebook. Disagreements were resolved through discussion and revisions of codes until consensus was reached. To elucidate the relationship between moral distress, hospitals’ institutional culture and structures, and societal factors in end-of-life care, all codes related to moral distress were extracted. T.D.B. analyzed these codes and generated initial themes. Themes were challenged with counterfactual data and further refined during iterative team discussions between T.D.B., E.W.D., J.N.B., S.W.G., and J.K.A. until consensus was reached. We used ATLAS.ti software version 24.2.0 (ATLAS.ti Scientific Software Development) to manage data. Data were analyzed in 2 phases, from January 2019 to December 2022 and from June to September 2024.

## Results

Table 1 presents the demographic characteristics of the 122 interviewees (75 physicians [61%]; 22 nurses [18%]; 6 advanced practice clinicians [6%]; 68 [56%] women; mean [range] age, 42 [27-74] years), including 25 Asian participants (20%), 6 Black or African American participants (5%), 7 Hispanic participants (6%), 2 Middle Eastern participants (2%), 9 multiethnic participants (7%), and 71 White participants (58%); 2 participants (2%) identified as other race or ethnicity or did not provide information. The mean and median interview length was 47 minutes (range, 30-60 minutes).

**Table 1.** Demographics of Interviewees

Characteristic	Respondents, No. (%) (N = 122)
Profession	
Physician	
Overall	75 (61)
By level	
Attending	57 (47)
Fellow	10 (8)
Resident	8 (7)
By specialty	
Emergency medicine	10 (13)
Ethics	5 (6)
Geriatrics	3 (4)
Critical care	29 (37)
Internal medicine	23 (29)
Palliative care	8 (10)
Advanced practice clinician	6 (6)
Nurse	22 (18)
Chaplain	3 (2)
Cultural mediator	3 (3)
Social worker	4 (3)
Race and ethnicity	
Asian	25 (20)
Black or African American	6 (5)
Hispanic	7 (6)
Middle Eastern	2 (2)
Multiethnic	9 (7)
White	71 (58)
Other or information not provided^a^	2 (2)
Gender	
Men	50 (41)
Women	68 (56)
Other or information not provided	4 (3)
Age, mean (range), y	42 (27-74)
Work experience, mean (range), y	15 (1-52)

^a^ Includes individuals who identified as other race or ethnicity or who did not provide information.

Overall, respondents felt moral distress could be exacerbated by a hospital culture of health care consumerism, medical hierarchies, and insufficient institutional structures to support clinicians’ efforts to de-escalate potentially nonbeneficial LST. Respondents felt moral distress could be prevented or mitigated by institutional policies to empower clinicians across the medical hierarchy, resources to address conflicts, and clinician-driven quality improvement (QI) initiatives and supportive hospital leadership.

### Factors Exacerbating Moral Distress

#### Health Care Consumerism

Respondents felt health system marketing and reputation affected clinicians’ practice patterns. Respondents believed advertisements and public-facing messaging encouraged use of LST (Table 2, quotation [Q] 1 and Q2). One respondent believed that a prominent hospital executive’s public remarks influenced hospital culture toward resisting death at all costs (Table 2, Q1). Another respondent felt their institution’s advertising of its cancer care was connected to oncologists’ willingness to administer cancer-directed therapies to patients who are terminally ill (Table 2, Q2). Increased use of potentially nonbeneficial LST and other high-intensity treatments was perceived as worsening clinicians’ experiences of moral distress (Table 2, Q1 and Q2).

**Table 2.** Illustrative Quotes for Institutional Factors Exacerbating Moral Distress Related to Potentially Nonbeneficial LST

Theme	No.	Quotation (location; respondent)
Health care consumerism	1	“Our previous [CEO] is very famous. [They] gave an interview, and they asked [them] about our health system. They said, ’At [hospital 2], we don’t let people die.’ Everyone still talks about it. This is the culture. We don’t let people die.” (hospital 2; palliative care physician 2)
	2	“We have a joke that our oncologists will give cancer therapy to patients who are already in the grave…. That’s what all the billboards are for in our community, for our cancer care.” (hospital 4; resident physician 1)
	3	“What’s the motto? ‘Re-defining possible.’ The expectation is that [hospital 1] will be able to solve the issues. Sometimes, those issues are unfortunately not solvable… as a provider, you’re setting yourself up for a lot of heartache, frustration, sadness…” (hospital 1; hospital medicine physician 2)
	4	“There are times when we can’t operate like a hotel or an amusement park or a restaurant because it’s not the right thing for the patient, and we have to tell them ‘no’ at times… it’s bad medicine.” (hospital 2; patient experience administrator 3)
	5	“The family says, ‘Keep coding them.’ You’re breaking their ribs. Blood is coming out of their mouth. But that’s what the family says they want. It’s very, very distressing. What is ethical? Doing what the family wants no matter what?” (hospital 2; ICU nurse 3)
Medical hierarchies constrained clinicians	6	“No one asked the nurses what their conversations were with the patient, or what their opinions were…. They felt like if there could have been more communication, they would have been able to advocate better for their patient…. This caused huge moral distress.” (hospital 2; ICU nurse lead 1)
	7	“[ICU leadership] doesn’t really help me escalate my conversation. It just kind of stops there…. Next day I come back, nothing has happened…. Another day in the ICU means suffering for the patient.” (hospital 1; neurology ICU nurse 3)
	8	“Our culture isn’t great about including nurses routinely. It takes a nurse getting fed up and standing up and making a strong statement.” (hospital 1; palliative care physician 2)
	9	“I feel some tension when I’m asked to care for the oncology patients…. I feel like I’m often asked to or pressured into providing care that would be more aggressive than I would otherwise do.” (hospital 2; ICU physician 1)
	10	“The family [will] be interested in more comfort measures or no escalation of care…. Then the oncology team will talk to them, and it will be full steam ahead despite having a very poor prognosis…. It feels frustrating because you feel like you’re doing something that’s futile and that the patient may not have wanted.” (hospital 2; hospital medicine physician 1)
	11	“The surgical specialties… have been just astoundingly frustrating…. You don’t have any control over what therapies they’re offering…. The family is asking you about things, and we don’t have any power…. It’s bordering on true cruelty to this patient.” (hospital 1; ICU physician 6)
Insufficient institutional structures to support treatment de-escalation	12	“He [the patient] was dependent on transfusions, dialysis, and tube feeds, and it was very unlikely that he was going to be getting better…. I would have appreciated some sort of policy or way to have the system help us because it just felt so impossible.” (hospital 1; palliative care physician 2)
	13	“[There] is always a new team to be consulted, a new salvage rescue therapy we can try. There’s a lot of momentum around adding and continuing treatments and there don’t seem to be as clear-cut opportunities or processes to discontinue or withhold potentially nonbeneficial treatments…. At some point, the emotional distress within the team starts to build momentum.” (hospital 1; ICU nurse 8)
	14	“It’s not that [ethics consultations] are ever unhelpful. Sometimes there’s really not much to do or say, really.” (hospital 3, ICU physician 4)
	15	“There’s a threat to engage risk [management]…. Talking about ethics… same thing with risk, I don’t feel like risk helps us to align with families.” (hospital 1, hospital medicine physician 1)

Abbreviations: CEO, chief executive office; ICU, intensive care unit.

Respondents felt marketing and institutional reputation shaped patients’ and families’ expectations. One respondent believed an institutional motto suggested to patients that the institution could always solve their medical problems (Table 2, Q3). The respondent explained how marketing and reputation could lead clinicians to feel disappointed and frustrated when they could not help patients achieve their desired outcomes.

A respondent described how their institution conceived of patients as consumers of a commercial product, like customers at a hotel or restaurant (Table 2, Q4). They perceived this mindset as harmful because it reflected hospital norms wherein clinicians felt unable to tell patients they would not benefit from certain treatments. Another respondent explained how deference to a family’s request for potentially nonbeneficial LST caused them moral distress, and they doubted whether this was ethical (Table 2, Q5). Respondents linked patients’ and families’ expectations regarding treatment intensity with increased use of potentially nonbeneficial LST, contributing to experiences of moral distress (Table 2, Q3).

#### Medical Hierarchies Constrained Clinicians

Medical hierarchies led nurse respondents to perceive that they could not affect the trajectory of potentially nonbeneficial LST, which compounded their moral distress. Nurses reported experiencing moral distress when physicians did not solicit their perspectives regarding whether treatments were medically beneficial or aligned with patients’ preferences (Table 2, Q6) and when physicians disregarded their concerns (Table 2, Q7). Other nurse respondents believed that for their perspectives to be considered in hospital cultures that systematically excluded them from medical decision-making, it was incumbent on nurses experiencing moral distress to challenge physicians, who occupied positions higher in the hierarchy (Table 2, Q8).

Primary team physician respondents (eg, hospitalists, intensive care unit physicians) also felt disempowered relative to subspecialty consultants (eg, oncologists, surgeons) in the medical hierarchy, which contributed to the former experiencing moral distress. Primary team physicians reported feeling constrained by subspecialty consultants, whom they understood to determine the trajectory of potentially nonbeneficial LST at their hospital (Table 2, Q9-Q11). Primary team respondents stated they sometimes felt pressured by subspecialty consultants to provide higher-intensity treatments that they believed were not medically beneficial (Table 2, Q9) and possibly discordant with patients’ preferences (Table 2, Q10). Primary team respondents also described feeling powerless to stop subspecialty consultants when the latter offered LST that might cause patients harm (Table 2, Q11). Primary team physicians felt frustrated when they perceived their clinical autonomy was undermined by subspecialty consultants (Table 2, Q10 and Q11). For nurse and primary team physicians, feeling constrained by other clinicians in the medical hierarchy (Table 2, Q6, Q7, and Q11) and complicit in delivering potentially nonbeneficial LST (Q7, Q9, and Q11) contributed to moral distress.

#### Insufficient Institutional Structures to Support Treatment De-Escalation

Respondents described feeling unsupported by the hospital system when patients were receiving potentially nonbeneficial treatments (Table 2, Q12). Respondents felt their institutions did not have policies (Table 2, Q12) or protocols (Q13) supporting decisions to de-escalate potentially nonbeneficial LST. Several respondents linked their inability to withhold or withdraw such treatments to experiences of moral distress (Table 2, Q12 and Q13). Some respondents stated that ethics consultants and risk management—resources intended to help clinicians navigate ethical challenges—were unhelpful in resolving morally distressing scenarios and could even paradoxically worsen clinicians’ relationships with patients and families by hindering alignment with the medical team (Table 2, Q14 and Q15).

### Factors Preventing or Mitigating Moral Distress

#### Institutional Policies Empowered Clinicians Across the Medical Hierarchy

Respondents viewed favorably policies granting nurses autonomy to independently consult palliative care and ethics teams when they experienced moral distress (Table 3, Q16 and Q17). Respondents reported these policies enabled nurses to circumvent conflict with physicians (Table 3, Q17). Respondents felt such policies helped produce a hospital culture of power-sharing across the medical hierarchy and encouraged teams to reconsider the trajectory of potentially nonbeneficial LST contributing to moral distress (Table 2, Q16).

**Table 3.** Illustrative Quotes for Institutional Factors Preventing or Mitigating Moral Distress Related to Potentially Nonbeneficial LST

Theme	No.	Quotation (location; respondent)
Institutional policies empowered clinicians across the medical hierarchy	16	“Palliative care can be consulted by anybody in the hospital…. We have a lot of power sharing among the teams at [hospital 3]. I think that [has] empowered nursing…. Sometimes that prompts teams to have conversations that needed to be had. Sometimes that means it’s an expression of moral distress.” (hospital 3; palliative care physician 1)
	17	“Before, we didn’t have any nurses consulting ethics because they were scared of physicians yelling at them…. [Now] nurses don’t have to get the physician’s permission…. You’re feeling an ethical dilemma. You’re feeling moral distress. What do you do? Now they can always consult ethics.” (hospital 4; ICU nurse 1)
Institutional resources addressed conflicts and provided emotional support	18	“[The patient] was from East Africa…. We just needed to get everybody together to come to some understanding that the patient had reached the end of the line, and it was time to respectfully let him go. And it was helpful to have the cultural mediator there to facilitate not just the language translation from Amharic, but the other general cultural issues about family beliefs.” (hospital 3; ICU physician 4)
	19	“[In] palliative care, we often get consulted because… there’s moral distress among providers. It feels like there’s a right thing to do medically and that’s different from what the family or the patient is choosing…. [We] listen to both perspectives, giving the providers a chance to express the distress they are feeling and giving the family or the patient a chance to explain who they are and what their beliefs and values are.” (hospital 3; palliative care physician 4)
	20	“We also have a death round once a month…. It’s really directed at the interns…. It’s meant to deal with the emotional stress of taking care of people in the ICU…. Sometimes, it’s related to the discomfort people have providing care that they’re not comfortable with or they think is inappropriate; that is stressful.” (hospital 3; ICU physician 4)
	21	“The social worker and chaplain spiritual services started this workshop that was offered to nurses. You could go there and share anonymously your experience and what is causing burnout [and] moral distress…. They taught very helpful strategies to address burnout, compassion fatigue.” (hospital 1; neurology ICU nurse 3
Clinician-driven quality improvement initiatives and supportive hospital leaders	22	“We introduced this tool into the initial documentation…. It was integrated as a score: high, moderate, and low risk for [an] ethical dilemma…. It allowed us to integrate with our liver transplant team because it had to be part of our team rounds…. [W]hich was great because [the nurses] learned a different language. And so did our physicians, which was a big leap for our service… if [the risk score] was high enough, it got escalated to physician leadership.” (hospital 2; ICU nurse lead 2)
	23	“I did an audit: out of 30 family meetings about goals of care and end of life, we had 3 nurses who attended. I took that data and presented it to the leadership, and I got support…. Moving forward, we made it mandatory… if it’s [a] family meeting, the bedside nurse will attend. Mandatory.” (hospital 4; ICU nurse 1)
	24	“We are looking to avoid care we think is not medically effective, and we’re more deliberate than some other places where care does become extremely aggressive…. Palliative care deserves credit for this [culture]…. It’s all these layers. [Clinician, name redacted]’s work with advanced care planning notes…. The work we did in the ICU redesigning our comfort care guidelines… [a different clinician, name redacted]’s work and the influence she’s had on nurses throughout the institution. Interprofessional rounding in the ICU…. So, I think the culture comes from a lot of different places.” (hospital 1; ICU physician 1)

Abbreviation: ICU, intensive care unit.

#### Institutional Resources Addressed Conflicts and Provided Emotional Support

One hospital employed cultural mediators, non–clinically trained practitioners who helped address challenges among clinicians, patients, and families arising from discordances in individuals’ linguistic and cultural backgrounds (eg, national origin, religion). Respondents felt cultural mediators were an important institutional resource for improving communication and developing mutual understanding among clinicians, patients, and families (Table 3, Q18). Cultural mediators enabled clinicians to better understand patients’ and families’ culturally informed values and beliefs and communicate why they believed LST were nonbeneficial or goal-discordant (Table 3, Q18).

Respondents identified palliative care teams as another institutional resource clinicians used when concerned about potentially nonbeneficial LST. Palliative care teams facilitated opportunities for patients and families to discuss their values and preferences with the medical team (Table 3, Q19). Palliative care teams and ethics consults also served as important outlets for some clinicians to express their moral distress (Table 3, Q16, Q17, and Q19).

Respondents described debriefing and emotional support resources, including workshops or death rounds (ie, organized discussions related to end-of-life care) as providing structured forums for clinicians, particularly nurses and trainee physicians, to discuss challenging experiences, such as emotional stresses of caring for patients (Table 3, Q20), burnout, compassion fatigue, and moral distress (Q21). Respondents reported that debriefing and emotional support resources taught participants techniques for managing these challenging experiences and emotions (Table 3, Q21).

#### Clinician-Driven QI Initiatives and Supportive Hospital Leaders

One respondent recounted how their department incorporated an ethical risk score into multidisciplinary rounds, which they felt improved communication across—and mitigated constraints imposed by—the medical hierarchy (Table 3, Q22). Using this tool, a protocol was created for automatically escalating high-risk ethical situations to hospital leaders (Table 3, Q22). Another respondent conducted an audit and found that nurses on their unit rarely participated in goals of care discussions (Table 3, Q23). With leadership support, they changed unit policy to require nurse attendance at family meetings (Table 3, Q23).

A third respondent praised the positive influence of palliative care and described how individual policies, protocols, and resources created a hospital culture wherein clinicians were more deliberate in their use of LST and were supported when they considered withholding or withdrawing potentially nonbeneficial LST (Table 3, Q24). Respondents highlighted the importance of the individual clinicians who championed these issues and initiatives coupled with the support of departmental leadership in driving institutional structural and hospital cultural change related to potentially nonbeneficial LST (Table 3, Q23 and Q24).

## Discussion

In this qualitative study conducted at 4 hospitals, clinicians perceived that hospitals’ institutional culture and structures affected clinicians’ experiences of moral distress related to potentially nonbeneficial LST. Institutional factors appeared to affect moral distress by exacerbating, preventing, or mitigating the influence of interrelated societal factors: defaults of high-intensity treatments, health care consumerism, and medical hierarchies (Figure). These results align with previous findings that hospitals’ institutional culture and structures influence trajectories toward or against the default of high-intensity treatments near the end of life.^8,10,32,45^ This study found health care consumerism and medical hierarchies were viewed as mutually reinforcing societal factors that clinicians believed could constrain attempts to de-escalate potentially nonbeneficial LST and contribute to moral distress. These results highlight the importance of developing tailored institutional-level interventions (eg, policies, resource allocation, attention to hospital culture) to prevent or mitigate the impact of societal factors on moral distress related to potentially nonbeneficial LST.

**Figure.**
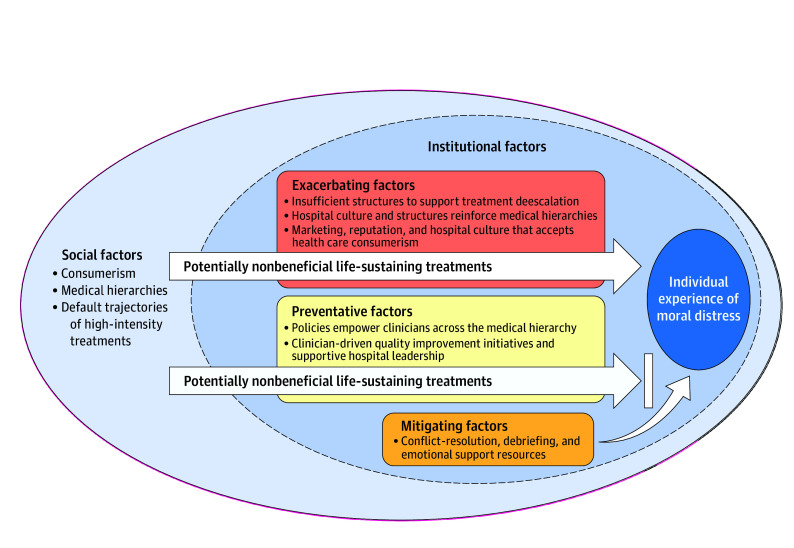
How Institutional and Societal Factors Can Affect Moral Distress Related to Potentially Nonbeneficial Life-Sustaining Treatments

Clinicians perceived that health care consumerism influenced care and contributed to moral distress. Consumerism counters medical paternalism by advocating that consumers (ie, patients) should have control over their health care decisions.^50^ Critics argue consumerism encourages commodification of health care and erosion of physicians’ professional integrity by denying them the ability to refuse requests for potentially nonbeneficial treatments.^28,31,51,52^ Clinicians in our study echoed this perspective, describing hospital cultures that viewed patients as consumers whose preferences superseded clinicians’ recommendations. When clinicians felt unable to decline patients’ and families’ requests for potentially nonbeneficial LST, they reported experiencing moral distress.

Medical hierarchies—a component of many definitions of moral distress^15^—were another societal factor that clinicians believed could lead to moral distress. Nurse respondents reported moral distress related to their exclusion from medical decision-making, a dynamic widely described in the literature^33,53,54^ that may further nurses’ structural disempowerment.^55^ Our results highlighted another less well-recognized element of the medical hierarchy: power imbalances between primary team and subspecialty consultant physicians. Whereas physicians are unambiguously above nurses in traditional medical hierarchies,^34^ power differentials between physicians of different specialties can be less clear.^35,36,37,38^ Primary team physicians described sometimes feeling constrained by subspecialty consultants and experiencing moral distress when consultants set a clinical trajectory involving potentially nonbeneficial LST.

Our results demonstrate how institutional factors might contribute to moral distress by exacerbating the mutually reinforcing societal factors of defaults of high-intensity treatments, health care consumerism, and medical hierarchies. Institutions may lack sufficient structures to support clinicians’ efforts to de-escalate trajectories toward potentially nonbeneficial treatments. Marketing and resource allocation decisions (eg, funds directed toward cancer centers) might bolster a health care system’s reputation as an institution where patients and families could expect to receive high-intensity treatments, even when potentially nonbeneficial. This dynamic may reflect the constraining influences of consumerism on hospitals’ institutional culture and structures.^28,31^ Marketing, reputation, and resources could contribute to a hospital culture encouraging specialist physicians to administer potentially nonbeneficial treatments and disempowering primary team physicians within an informal medical hierarchy.

Another important finding is how institutions might prevent or mitigate societal and institutional contributors to moral distress. Permitting nurses to consult palliative care and ethics teams allowed nurses to participate in decision-making, reducing their perceived powerlessness and consequent moral distress. Such policies may enable institutions to leverage limited resources toward root causes of moral distress. Notably, whereas physicians may technically be able to de-escalate nonbeneficial LST, they felt pragmatically constrained by insufficient institutional structures to support efforts to de-escalate treatments.

In contrast to nurse-physician formal power imbalances, different approaches may be required to address informal hierarchical constraints that primary team physicians experienced vis-à-vis their subspecialty consultant colleagues. Strategies solely seeking to improve team-based communication^26^ without considering institutional factors exacerbating informal hierarchies may yield limited results. Our results suggest that, beyond the immediate impacts of any specific intervention, the process of identifying, designing, and implementing policies to address structural sources of moral distress appears to empower clinicians. Hospital leaders could consider ways to support clinician-driven QI initiatives to improve policies and create a hospital culture more responsive to clinicians’ concerns.

Increased palliative care team support may prevent or mitigate moral distress. Respondents felt palliative care teams mediated conflicts among patients, families, and clinicians; provided clinicians with debriefing and emotional support after distressing events; and developed policies and influenced hospital culture to prevent and reduce the provision of potentially nonbeneficial LST. Careful attention should be directed toward the potential financial and ethical ramifications for patients and clinicians if palliative care teams’ responsibilities expand to include addressing moral distress.^56^ Alternatively, dedicated moral distress consultation services could support clinicians.^57^

Finally, our results highlight the importance of tailored institution-specific interventions to target institutional contributors to moral distress related to potentially nonbeneficial LST. Respondents at a hospital serving a diverse, underserved patient population noted the role of cultural mediators in addressing challenges among clinicians, patients, and families arising from discordances in individuals’ linguistic and cultural backgrounds, which may have influenced preferences for potentially nonbeneficial LST.

### Limitations

While additional societal and institutional factors may influence clinicians and institutions, we could not explicitly investigate those factors beyond respondents’ general perceptions that they affected moral distress. Our observations were based on 4 urban, highly resourced academic medical centers and may not generalize to rural, lower-resourced, or nonacademic hospitals.

## Conclusions

In this qualitative study, we described institutional factors that may exacerbate, prevent, or mitigate the influence of societal factors contributing to moral distress related to potentially nonbeneficial LST. Health systems should consider how health care consumerism influences patients’, families’, and clinicians’ expectations regarding potentially nonbeneficial LST, particularly at hospitals with advanced technological interventions (eg, organ transplantation, extracorporeal membrane oxygenation, salvage chemotherapies). Future studies should explore the societal and institutional factors contributing to moral distress for clinicians at lower-resourced hospitals, such as inaccessible advanced treatments and barriers to transferring patients for higher levels of care. More research is needed to better understand how formal and informal medical hierarchies (eg, nurse-physician, primary team-subspecialty consultant) shape moral distress.

While some institutions lacked sufficient structures to support clinicians’ efforts to de-escalate potentially nonbeneficial treatments, policies empowering clinicians across the medical hierarchy, as well as conflict resolution and emotional support resources (eg, palliative care) might prevent or mitigate moral distress. Hospital leaders should support clinician-driven QI initiatives to improve policies regarding potentially nonbeneficial treatments and foster a hospital culture responsive to clinicians’ concerns. This study highlights the importance of tailored institutional-level interventions to address institutional contributors to moral distress related to potentially nonbeneficial LST.

## Notes

Supplement.
Data Sharing Statement
